# Pushing the science forward: chitosan nanoparticles and functional repair of CNS tissue after spinal cord injury

**DOI:** 10.1186/1754-1611-7-15

**Published:** 2013-06-03

**Authors:** Bojun Chen, Debra Bohnert, Richard Ben Borgens, Youngnam Cho

**Affiliations:** 1 Center for Paralysis Research, Department of Basic Medical Sciences, College of Veterinary Medicine, Purdue University, West, Lafayette, IN 47907, USA; 2 Weldon School of Biomedical Engineering, Purdue University, West, Lafayette, IN 47907, USA; 3 Present address: New Experimental Therapeutics Branch, National Cancer Center, 809 Madu-1dong, Ilsandong-gu, Goyang-si, Gyeonggi-do, 410-769, Korea

## Abstract

**Background:**

We continue our exploration of the large polysaccharide polymer Chitosan as an acute therapy for severe damage to the nervous system. We tested the action of subcutaneously injected nanoparticles (~ 100 – 200 nanometers in diameter; 1 mg per ml) against control injections (silica particle of the same size and concentration) in a standardized *in vivo* spinal cord injury model. These functional tests used standardized physiological measurements of evoked potentials arriving at the sensorimotor cortex subsequent to stimulation of the tibial nerve of the contralateral hindlimb. We further explored the degree of acetylation and molecular weight of chitosan on the success of sealing cell damage using specific probes of membrane integrity.

**Results:**

Not one of the control group showed restored conduction of evoked potentials stimulated from the tibial nerve of the hindleg – through the lesion – and recorded at the sensorimotor cortex of the brain. Investigation if the degree of acetylation and molecular weight impacted “membrane sealing” properties of Chitosan were unsuccessful. Dye - exchange membrane probes failed to show a difference between the comparators in the function of Chitosan in ex vivo injured spinal cord tests.

**Conclusions:**

We found that Chitosan nanoparticles effectively restore nerve impulse transmission through the crushed adult guinea pig spinal cord in vivo after severe crush/compression injury. The tests of the molecular weight (MW) and degree of acetylation did not produce any improvement in Chitosan’s membrane sealing properties.

## Background

The integrity of the cell membrane is critical for maintaining cell physiological structure and function. For example, immediate function loss, progressive degeneration, and the death of neurons after acute spinal cord injury (SCI) is initiated after cell membrane disruption [1-4]. Spontaneous membrane self-repair is often initiated after the damage, but fails to overcome the overwhelming tissue distortion and physiological derangement, such as unregulated Ca^2+^ influx, reactive oxygen species (ROS) generation, and subsequent lipid peroxidation (LPO) (reviewed in [5] and [6]). Among all therapies, restoring membrane integrity rapidly and effectively after injury would be critical in early stages of Central Nervous System (CNS) damage interfering with progressive secondary injury [5,7-9].

It has been established that water-soluble polymers such as polyethylene glycol (PEG) can fulfill some of the requirements discussed above. PEG and some other synthetic polymers rapid and effectively seal membrane disruption [2,10]. Moreover it can preserve very significant structural, physiological, and behavioral function after SCI, Traumatic Brain Injury (TBI), and even localized peripheral nerve damage [2,11-13]. However, due to the viscosity of high molecular weight (MW) and the toxicity of low MW after the degradation of PEG, its administration must be limited in concentration and in timing after acute clinical neurotrauma [14,15]. Under some circumstances PEG can be quite toxic to CNS tissue [16-19] and see Figure Thirty one; Recovery of Behavioral and Physiological Function In Vivo page 128 in Borgens 2003 [20]. In a parallel line of investigation, studies showed that Chitosan also had similar and even more significant sealing actions than PEG [21]. Actually, the effect of chitosan, a non-toxic biodegradable polysaccharide polymer, has already been widely studied and used in biomedical and industrial applications, such as beverage clarification, wound healing, surgical adhesion, and drug delivery [22,23].

**Figure 1:**
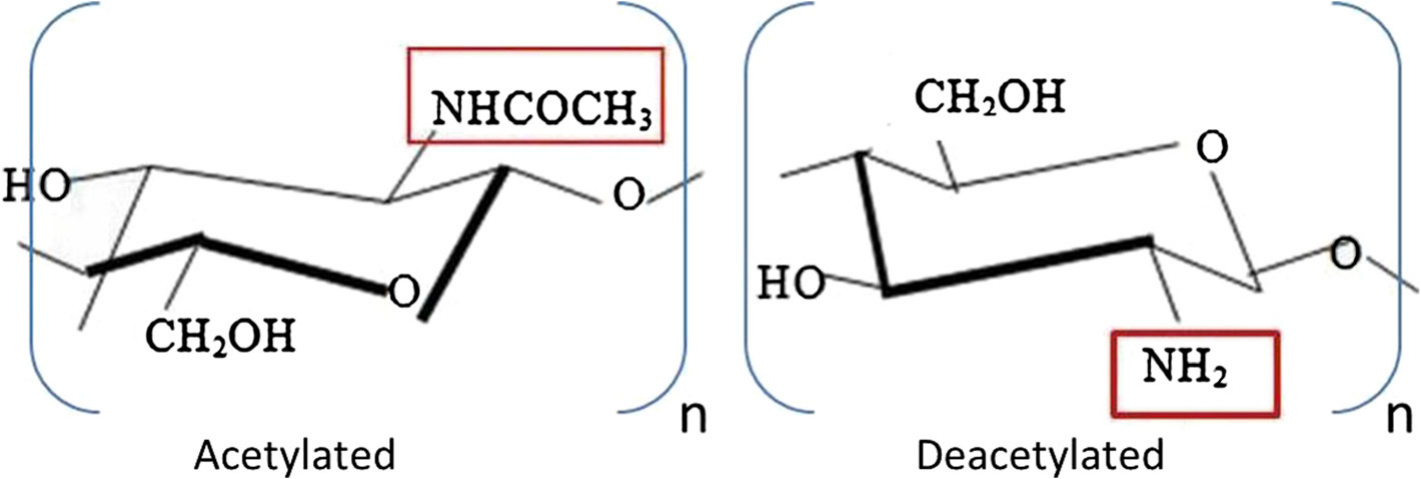
**The chemical composition of acetylated and deacetylated chitosan. **The amine group of the deacetylated chitosan (on the right), squared in red, is produced by the removal of the acetyl group, -COCH_3_, from the acetylated chitosan (on the left) during the process of the deacetylation. Therefore, the amine group, squared in red, in the deacetylated chitosan can be protonated in the acidic environment.

The chemical characteristics of Chitosan are mainly determined by two variables: the degree of acetylation (DA) (Figure 1) and MW [24]. The DA determines the number of free amino groups in the chitosan polymer which is inversely proportional to the degree of protonation. On the other hand, the MW determines the length of the main chain of the polymer, which potentially influences the viscosity of the solution and the shape of the polymer when it is presented in solution to targets such as cells and tissues. Previous studies showed that the DA plays a critical role in artificial membrane incorporation, mammalian membrane sealing, and neuro-physiological function restoration ex vivo [21,25-28]. Electrostatic interaction between negative charged lipid headgroups on the cell membrane and the primary amines on the cationic deacetylated unit, β-(1–4)-linked D-glucosamine on the chitosan backbone has been proposed as the major physical driving force for its membrane adsorption and incorporation [25-27].

The effect of hydrophobic interaction between the inner non-polar hydrocarbon chain of the membrane and the nonionic acetylation unit, N-acetyl-D-glucosamine on the backbone of chitosan is also claimed to complete membrane insertion after chitosan adsorption onto the membrane surface [25-27]. As mentioned above, MW is closely related to the morphological structure of chitosan presented in the aqueous solution. Previous studies suggested the important role of viscosity and physical shape of chitosan presented in chitosan aqueous solution in biomedical applications [29]. Furthermore we have learned that chitosan in an injectable solution moves throughout the systemic circulation – apparently without issue dependent on viscosity [21]. Thus the route of administration (Intraperitoneal, Intraveneous, or Subcutaneous) does not appear to matter in a manner similar to PEG [10,12]. Nanoparticles and microparticles offer increased targeting to damaged cells coincident with a corresponding decrease in the concentration of the polysaccharide circulating within the body. Whether chitosan nanoparticles are able to reach and repair damaged tissues as has chitosan in aqueous solution in spinal injured animals with vascular trauma has not been tested until this report.

In addition, the effect of MW of chitosan on membrane organization has been discussed, mainly in the field of artificial lipid membranes considered as a simplified cell model. However, the issue was not settled [28,30,31]. Fang reported the enhanced interactions of lipid bilayers with higher MWs of chitosan, indicated by the significantly reduced cooperative units compared with those of lower MWs [30]. In contrast, Quemeneur observed the independent role of chitosan’s MW on membrane adsorption in controlled pH environments [28].

Using these observations, we push our laboratory’s success using Chitosan as an acutely applied membrane “sealant” forward – and present the first functional test of chitosan nanoparticles (100 – 200 nm diameter) application by subcutaneous injection to adult guinea pigs with crush/compression injury to the mid-thoracic spinal cord.

The use of nanoparticles (> 100 nm) provides a means to deliver Chitosan in large concentrations to only the interface of damaged cells (of course making up damaged tissues) escaping the need to systemically deliver the polymer as we have past reported. By revealing a functional benefit to nanoparticle administration – we further tested the “membrane repair” activity of Chitosan possessing different degrees of acetylation and molecular weight to determine the best composition/fabrication to move forward with further clinical and clinically oriented studies (Table 1). These studies used chemically modified chitosan in solution in tests of membrane integrity. These tests were: A) evaluating the loss of a large enzyme from cytoplasm to the extracellular milieu; and B), the intrusion of a dye molecule into the cytoplasm through compromised membrane. A more extensive discussion and technical detail relative to these “dye exclusion” techniques is detailed in [5,32]. Altogether, we pursue this line of investigation to provide a biodegradable alternative to PEG’s use following acute Neurotrauma.

**Table 1:** Different degree of acetylation and molecular weights of chitosan in this study

		**Samples**	**DA (%)**	**MW (Da)**
Molecular Weight (MW)	1 Low	Low MW chitosan	15 ~ 25	100 K
	2 Medium	Medium MW chitosan	15 ~ 25	200 K
	3 Oligo	Oligo MW chitosan	15 ~ 25	5000
Degree of Acetylation (DA)	4 DAO	DAO Chitosan	0	
	5 DA10	DA10 Chitosan	10	
	6 DA20	DA20 Chitosan	20	
	7 DA100	DA100 Chitosan	100	

## Discussion and conclusion

We provide the first preliminary results in the “whole animal” suggesting subcutaneous injection of Chitosan nanoparticles can dramatically improve nerve impulse conduction through the lesion – and upwards to the cerebral sensory centers after severe crush of mid-thoracic spinal cord of the adult guinea pig. In the absence of this experimental therapy, conduction remained silent. To our knowledge this is the first study of chitosan nanofabrications in any in vivo neurotrauma model. These data fit nicely with a previous in vitro investigation revealing a profound interference with progressive secondary injury mechanisms in acrolein - poisoned cell samples, membrane sealing, and the unique ability of chitosan particles to carry a “drug cargo”. This significantly enhances its therapeutic potential [21].

Our results reveal that Chitosan nanoparticles can indeed function as an important intervention in acute spinal cord injury by ameliorating the effects of acute compression/crush to the adult guinea pig spinal cord. The physiological recovery of conduction shown here frames the basis for functional recovery of motor, sensory, and autonomic functioning after neurotrauma (see discussion in [6]). However, being cautious, this promise is not yet fulfilled until clinically meaningful recovery of behavioral outcome measures is shown. Furthermore - by evaluating different compositions of chitosan in controlled solutions ex vivo - we do not believe MW or DA may be a critical factor in developing this use of a safe biodegradable polysaccharide for clinical treatment of SCI or TBI [12]. In other words, the strong membrane sealing effect of chitosan on damaged spinal cord tissues occurred regardless of DA and MW. Specifically, the TMR and LDH study confirmed the significant effect of chitosan on inhibiting i.) the intrusion of an exogenous applied dye into damaged spinal cord cells (P < 0.05); ii.) as well as escape of a large endogenous enzyme LDH through membrane defects (P < 0.05).

### Membrane sealing and repair

While there may be differences in scientific nomenclature between repair and sealing – those differences are moot in the context of trauma. The exchange of ions and macromolecules after membrane compromise is actually the initial event triggering the eventual death and degeneration of cells after injury (reviewed by [5,6]). The only logical means of acutely dampening and even reversing progressive secondary injury, cell degeneration, localized axotomy, and the demise of neurons and their support cells during the steady collapse of the nervous system is *to restore membrane integrity*, *at a minimum*, *to block this exchange*. This can be accomplished using various polymers and surface – active agents we have called membrane “sealants”. While the mechanisms of action may vary somewhat between inorganic polymers and polysaccharides while accomplishing this feat [32] – the outcome is the same: the membrane defect is acutely “plugged”; the organization of water and ions in and around the defect is normalized; and spontaneous reassembly of the plasmalemma then proceeds via lipid bilayer components resolving into one another. In the case of Polymers such as PEG – there is an affinity for damaged membranes based on the disruption of the hydrophilic outer leaflet. The membrane fusion is believed mediated by a dehydration effect and volume-exclusion aggregation of membrane lipids bringing adjacent lipids into close physical contact [2,3,10-13,33]. With Polymers such as Chitosan – the “reorganization” of membrane structure is initiated, but not limited to, the edge of the membrane defect. The subsequent continuing aggregation of chitosan on the membrane surface results from the extended conformation of chitosan’s main chain [25,26,28,30-32]. We hasten to add that PEG can facilitate a functional reconnection between proximal and distal segments of severed axons [2,20]. This takes many minutes of absolute immobility of the axon segments in the presence of the polymer to accomplish this task, and an unknown, and untested, period of immobilization of the tissue for the repair to become permanent. We do not see why chitosan would not as well induce membrane fusion permitting reconnection of axon segments in an experimental setting. However, in the context of the animal or human suffering from neurotrauma – such immobility would be impossible so this mechanism of action for any polymer-based sealant is a moot point.

### The issue of the degree of acetylation

The different degrees of acetylation represent different strengths of electrostatic interaction between the cationic chitosan and anionic lipids. Specifically, the lower the DA, the higher the number of positive ions a chitosan molecule carries. In turn, the higher concentration of surface charges on chitosan enables a more extended conformation due to electrostatic repulsion among charged amines on the chitosan backbone. Therefore, the polarity of the molecules is greatly enhanced. Since the lipid headgroups are negatively charged, molecules with lower DA might have a greater opportunity to diffuse close to the membrane breaches, and thus adsorb and seal the membrane through electrostatic interactions. This mechanism is consistent with previous studies in which chitosan polymers incorporated with artificial membrane films and vesicles where physical attractions and interactions were a result of membrane adsorption [25,26]. Our data however, did not reveal a hint of an improvement in function of chitosan based on the DA.

### The issue of molecular weight

Here, equivalent sealing effects using different MW chitosan treatments was suggested in both TMR and LDH tests, and in both transection and compression ex vivo injury models. Based on the fact that different MW chitosan in our study shared a similar DA, one possible reason for the consistency of data found in different MW chitosan treatments might be the predominant role of DA over chitosan MW in initiating membrane repair. A similar result is also observed in the zeta-potential evaluation of the influence of different MWs of chitosan on membrane adsorption [28].

**Figure 2:**
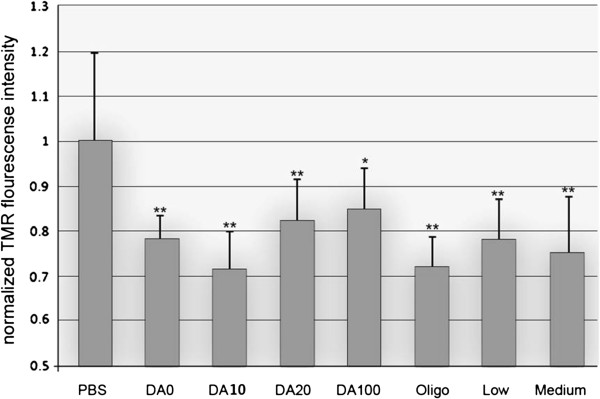
**Quantification of normalized fluorescence intensity of TMR uptake as a function of chitosan treatments. **The fluorescence intensity in the PBS group was used as the control. Note that all chitosan treatments significantly decreased the fluorescence intensity of TMR uptake following the transection injury, *P < 0.05, ** P < 0.01 (Dunnett test). A Statistical difference was not observed between chitosan treatments with different DA and MW, P > 0.05 (Tukey test). N = 6.

**Figure 3:**
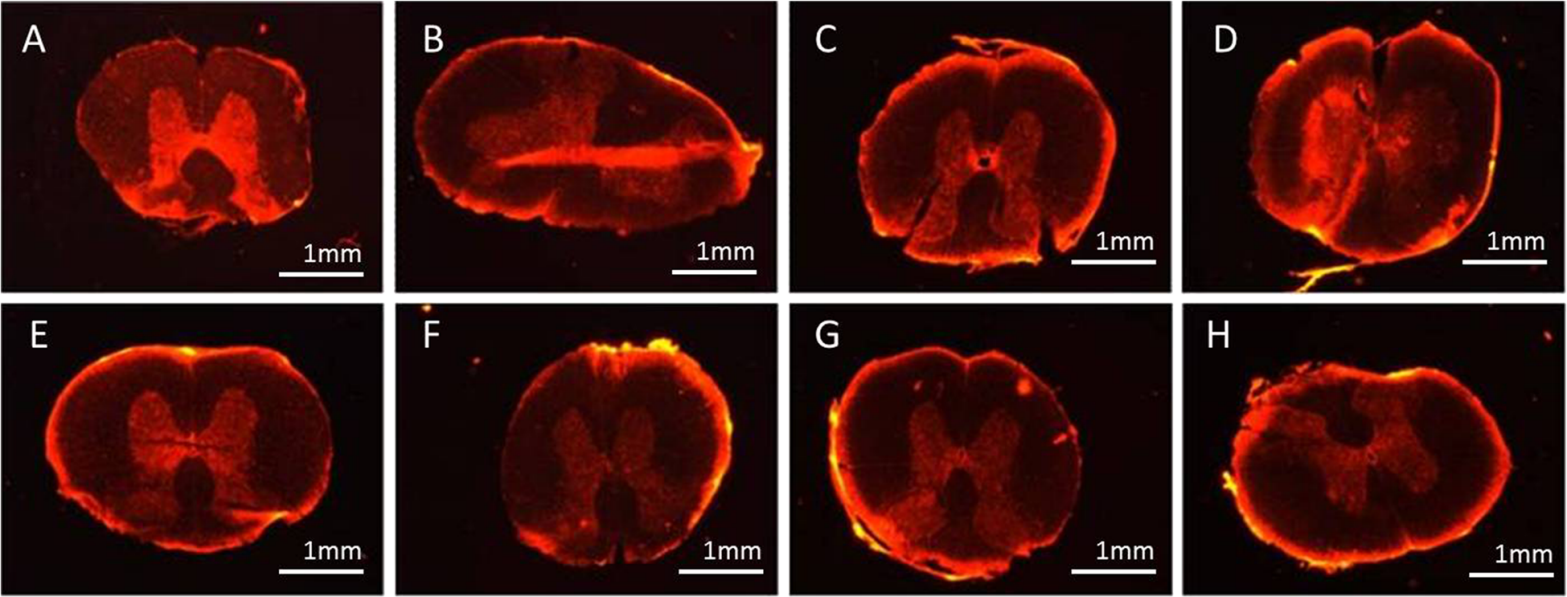
**The fluorescence intensity of TMR uptake after transection injury and its inhibition by different chitosan treatments. A**) control, **B**) DA100, **C**) DA20, **D**) DA10, **E**) DA0, **F**) Oligo, **G**) Low, **H**) Medium. See test for nomenclature.

The data shown in Figure 2 reveals that all tests showed a statistically significant reduction in TMR fluorescent intensity. There was little difference between them however - with the exception of DA100 which revealed a possible lower capability for sealing. An alternative thought is that the MW could play a role in membrane sealing [30,31] as it is closely related with the morphological structure of chitosan presented in the aqueous solution. Typically, the two most common structures are 1.) rope-like and 2.) coil-like polymeric shapes. The most likely shape in an oligo-chitosan solution would be rope-like due to the relatively few numbers of monomers existing in the chitosan. The less folded main chains of this polymer require less energy to diffuse close to the cell membrane and fit near or into its breaches. On the other hand, MW is also proportional to the viscosity of the aqueous solution, which reflects the hydrodynamic diameter of chitosan molecules; refer back to Figures 3 and 2[29]. The degree of the intermingling entanglement of chitosan polymer is proportional to the chain length, in other words - the MW. Considering the higher MW of Medium chitosan which is 40 times the size of the oligomer, this might result in the reduced mobility of the polymer. This is likely to enhance polymer adhesion by increasing the contact area between chitosan molecules and lipids [34].

### “Nanomedicine”

The superiority of nano-fabrications in particular over aqueous suspensions of injected polymers, cannot be under appreciated. High molecular weights of PEG are too viscous for facile IV use. Lowering the MW in an effort to produce a clinically easy injection may produce some level of toxicity due to the circulation of polymers which do not degrade and must be removed at the level of the kidney and liver (as discussed and cited above). Chitosan, as a naturally occurring polysaccharide, is not toxic. Detection of it in bodily fluids after systemic administration is unlikely.

Moreover, PEG coated silica nanoparticles required the PEG surface coat fabrication over the silica nanoparticle which is inert. To be further useful as a targeted repair agent and a delivery vehicle requires a third step - the bonding of surface PEG to the drug or cytokine of choice. In chitosan nanofabrications, the chitosan itself is the “sealant” while capturing or surface bonding of a drug/cytokine to be released requires only another single step in the process. Chitosan nanospheres - by themselves - show significant sealing properties producing improved physiological recovery after SCI.

## Results

### In vivo testing

#### SSEP responses to injection of chitosan microspheres

**Figure 4:**
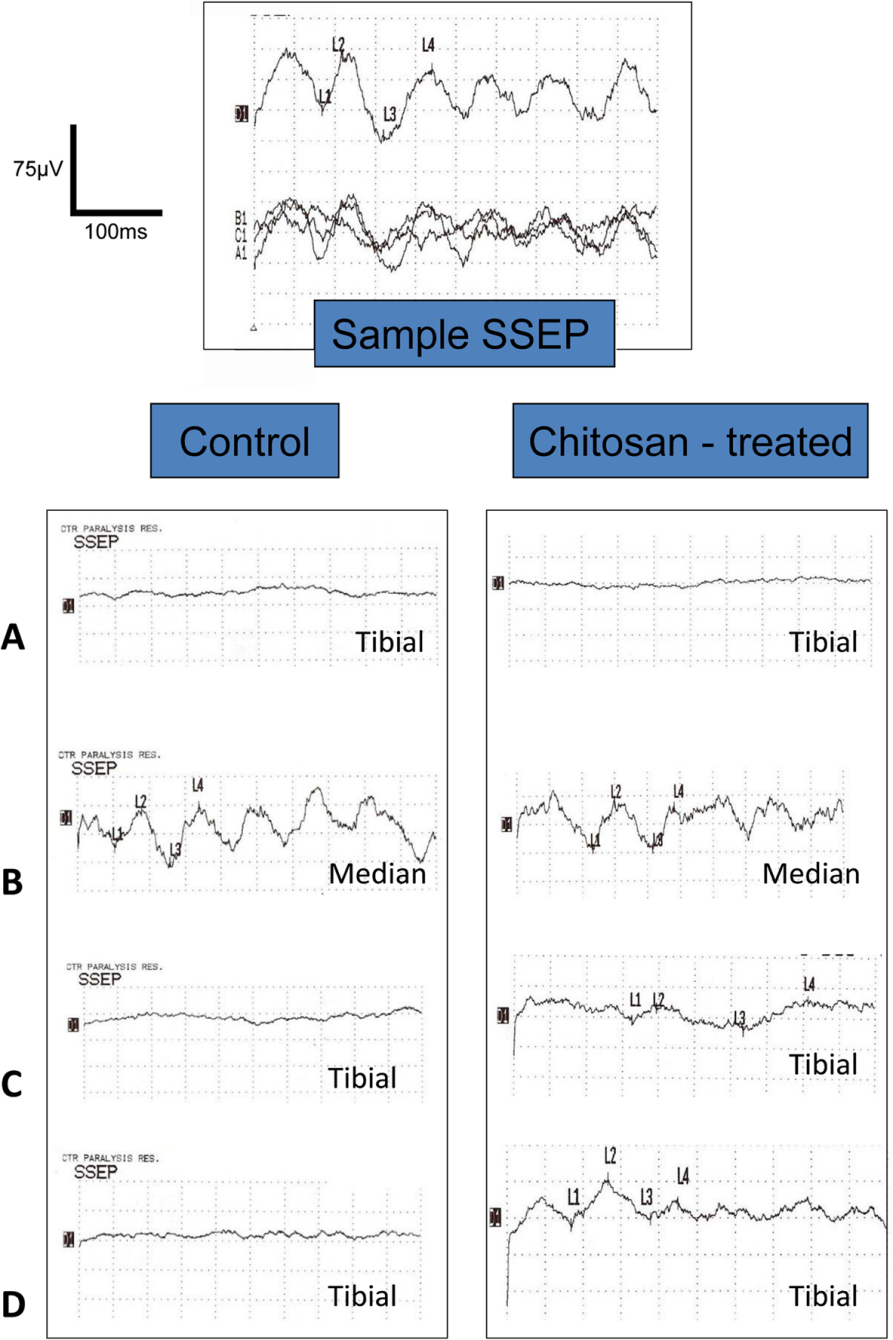
**Somatosensory recordings in control and injured guinea pig spinal cords. **The uppermost electrical record is a sample to show the single and averaged evoked potential as they are recorded. This record was produced by stimulation of the tibial nerve and recording the arrival of the ascending evoked potentials (EPs) of ~ 40 msec latency at the sensory motor Cortex. The three bottom traces are single records taken consecutively from the same uninjured animal as detailed in the text, while the top record is an average of these three. Any statistical deviation from baseline is automatically annotated. All data presented below are such averaged signals. The left column shows records taken from a Control animal. The right column shows records obtained from a Chitosan Nanoparticle-treated animal. Note that in both samples, compression injury eliminated transmission of EPs through the cord. Immediately below, the clear recordings of SSEP in both animals were obtained from Median Nerve stimulation/recording. This neural circuit from the forelimb to the sensory cortex is unaffected by injury to the midthoracic spinal cord, and is an internal control for the functioning of the system – eliminating the likelihood of “false negatives”. Note the appearance of weak EPs in only the Chitosan Nanoparticle – treated animal – while electrophysiological functioning in the Control animal were absent throughout the observation period. Reading down, recordings are; **A**) immediately post injury, **B**) median nerve control, **C**) one week post injury, and **D**) two weeks post injury. The time base is 10 msec per division, with an amplitude of 1.25 microvolts/division.

As expected, and observed many times before, all 5 silica nanoparticle – injected control animals failed to recover any form of evoked potential (EP) by 3 weeks after compression injury. The lack of conduction was confirmed by clear recordings of EPs elicited from Median nerve “control” stimulation at the same stimulation parameters before and after spinal compression. Of the 11 nanoparticle-treated animal group, 3 of these died prior to the final physiological measurement session. Instead of complicating evaluation by periodic scoring, we statistically compared only those animals that survived until the end of the study. SSEP conduction recovered in every one of these eight healthy animals. This difference between “controls and experimentals” was strikingly statistically significant (P = 0.007; Fisher’s exact; see Figure 4).

### Enzyme leakage from injured and untreated and treated spinal cords

**Figure 5:**
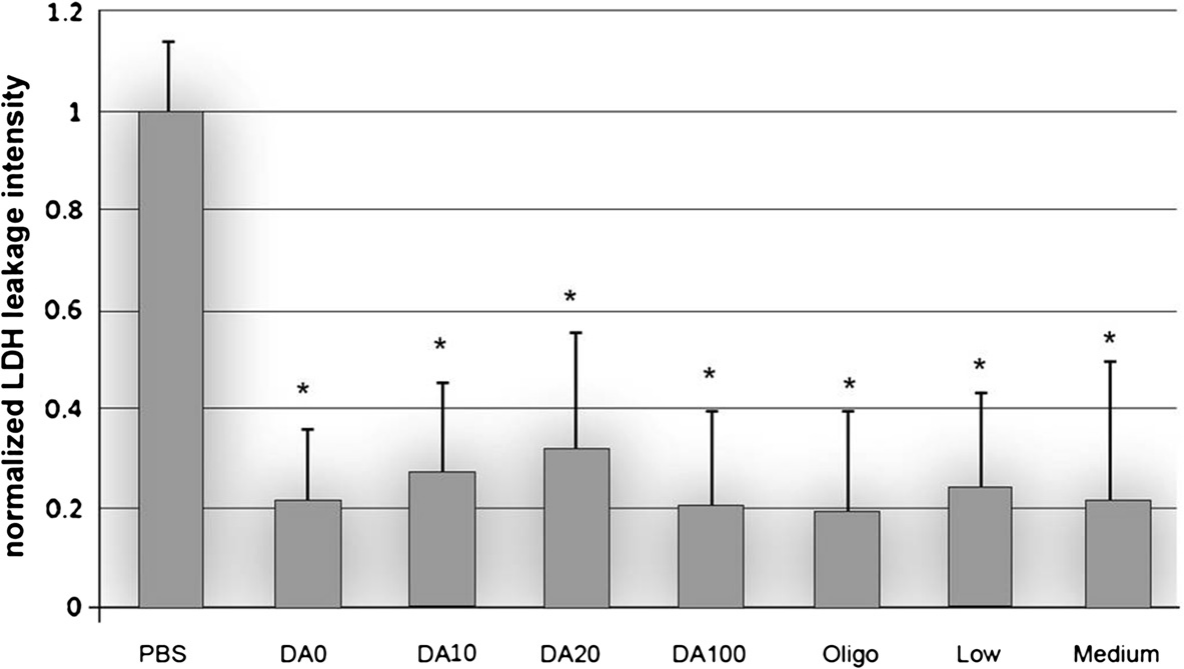
**Quantification of the normalized fluorescence intensity of LDH leakage as a function of chitosan treatments. **The fluorescence intensity in the PBS group was used as the control. Note that all chitosan treatments significantly decreased the fluorescence intensity of LDH leakage following the compression injury, *P < 0.05 (Dunnett test). A statistical difference was not observed between chitosan treatments of different DA and MW, P > 0.05 (Tukey test). N = 6.

The membrane sealing effect of chitosan was further confirmed by the inhibition of leakage of the intracellular enzyme Lactate Dehydrogenase (LDH) by specific assay (Figure 5). The signal intensity was proportional to the amount of LDH present in the bathing solution and inversely related with the condition of membrane integrity. More than a 60% decrease of the signal intensity after all chitosan treatments strongly indicated the sealing effect of chitosan (P < 0.05). Similar to the Tetramethylrhodamine (TMR) test, we did not observe any correlation with the result and the composition (MW and DA) of chitosan.

## Methods

### In vivo testing

#### Spinal cord injury in the adult guinea pig

We have used the adult guinea pig model of crush/compression injury to their mid-thoracic spinal cords for over 25 years. Extensive detail of the surgical manipulations and aftercare of spinal–injured guinea pigs, can be found in several previous reports (for examples see; [5,10,34,35]). Briefly: adult female guinea pigs (300 – 400 gms; Hartley strain) were deeply anesthetized, and a midline incision in the mid-thoracic regions exposed the vertebral column. A hemi-laminectomy procedure was performed exposing the spinal cord, and it was crushed using a laboratory - fabricated instrument which was basically a forceps possessing a détente. In this way the cord compression could be reasonably standardized between animals – and this “consultant displacement” injury is far superior in producing a standardized outcome than any “constant force”/weight drop technique. Animals were kept warm until fully recovered and moved to special housing where they received daily care from the technical staff of the Center for Paralysis Research (CPR) and Veterinary Animal Holding Facility.

#### The measurement of spinal cord evoked potentials in adult guinea pig as a measure of functional recovery

This laboratory pioneered the use of Somatosensory Evoked Potential (SSEP) recording using commercial clinical instruments for the measurement of evoked potentials as a means to determine conduction through guinea pig spinal lesions. We do not evaluate SSEPs to study the nature of subtle neurological conduction in CNS issues – but rather to determine the presence or absence of EP conduction relative to each animal’s preoperative recordings. For over a decade, we have learned this is the *most sensaive* of functional tests performed to judge the animals’ recovery from SCI. Sedated guinea pigs were connected to a Nihon Kohden Neuropack 2 Stimulator/Recorder using subcutaneous pin electrodes. Stimulating electrodes were inserted near the tibial nerve of the hindleg and a separate pair near the medial nerve of the ipsilateral foreleg.

The frequency of stimulation of the leg(s) was set at ~ 200 HZ which would produce a clear twitching of the very local region surrounding the stimulating electrodes. The magnitude of the stimulation was then reduced until such movement was nearly undetectable. Recording electrodes were inserted beneath the scalp superior to the region of the sensory cortex on the contralateral side (see the citations above for figures and for more detail). Control stimulation of the foreleg medial nerve was an excellent tool to prove electrode placement (and other variables) did not affect the recordings of ascending Evoked Potentials (EPs). This latter neural circuit was “above” the level of the injury to the cord. On the other hand, SCI completely blocks such conduction to the brain when the tibial nerve is stimulated. A recovery of EPs recorded at the contralateral sensory cortex subsequent to a standardized regimen of electrical stimulation of the hindlegs was taken as a recovery of electrophysiologi*c*al function in spinal cord injured animals. Recording of normal tibial – mediated SSEPs were always performed on each animal prior to SCI. Median nerve control procedures were a part of every recording period. Replacement or movement of electrodes was unnecessary since the medial nerve circuit set the standard for stimulation/recording, and the use of a laboratory – fabricated “toggle” allowed switching between stimulations of the hind and foreleg without touching the animal or the electrical connections.

#### Construction, evaluation, and administration of chitosan nanoparticles

We have already provided complete details of fabrication, chemical analysis, imaging, including the loading of drugs into nanoconstructions of Chitosan spheres as used in this study (for examples see [21,36]). We did not perform these analytical procedures again in this study since all features were well standardized, understood, and reported.

The Chitosan nanospheres used here were within 100 – 200 nm in diameter, fabricated using sterile media for use in animals, at a concentration of 1 mg of chitosan particles/ml of injection. 1 ml of this solution was injected subcutaneously in the nape of the neck, within ½ hour after SCI. Control injections of sterile lactated Ringers containing silica particles of the same dimensions and concentration were performed in a separate set of control animals accordingly. Only 5 control animals were studied since their use here was identical to over 50 such saline- injected animals used as controls in 4 individual published prior studies. Animals never recover SSEPs for varying periods of study durations up to – and sometimes exceeding - 1 month post SCI.

All guinea pigs used in this study were handled in accordance with, and prior approval by, the Purdue University Animal Care and Use Committee (PACUC).

#### Test solutions

0.1% (w/v) chitosan solutions completely dissolved in 1% acetic acid solution by stirring overnight. Figure 1 shows the acetylated and deacetylated forms of chitosan which is that of basic organic chemistry. Detail information on these polymers is provided in Table 1. All samples were bubbled with 95% O_2_/5% CO_2_ throughout the duration of the experiment.

#### Considering membrane permeability

The membrane permeability of damaged spinal cord segments with different treatments of different degrees of acetylation and molecular weights of chitosan solutions was evaluated by the interference with Tetramethylrhodamine (TMR; 4000 MW) and the leakage of the large endogenous enzyme Lactate Dehydrogenase (LDH, 140 kDa). The leakage of the relatively large molecular weight enzyme from disrupted membrane not only indicated compromize - but is a gauge of how severe such membrane breaches were [21,36]. The membrane sealing effect of chitosan was measured as a function of the leakage of LDH and the intrusion of TMR.

Briefly, after compression injury was induced, equally weighted segments were incubated immediately into different chitosan and 0.1 molL^-1^ Phosphate buffered saline (PBS) solutions respectively for 15 mins. Then, all segments were quickly rinsed in PBS solution for three times to remove any leaked LDH extracellularly. Segments were incubated in 1 ml fresh PBS solution for additional 45 mins to allow LDH leakage from un-repaired membrane breaches. The level of extracellular LDH in the above 600 μl bathing solution was assayed using the TOX-7 kit (Sigma-Aldrich, Inc, USA). Six replicates were applied in this study. Five measurements were taken for each sample.

#### Measurement of TMR uptake

The membrane sealing effect of chitosan was measured by the uptake of TMR. Briefly, immediately after transection injury, 1-cm segments of spinal cord were incubated in different chitosan solutions and PBS solution, respectively for 15 mins. Then, all segments were transferred and incubated in 0.1% TMR dextran solution for 15 mins in the dark. Subsequently, segments were quickly rinsed in 0.1 molL^-1^ PBS solution for three times and fixed in 4% paraformaldehyde for 5 h at 4°C in the dark. Segments were then embedded in Tssue –Tek OCT compound, frozen in liquid nitrogen. Sections were cut at 50 μm thickness using a freezing microtome (Thermo Electron, Waltham, MA, USA). Sections were visualized by epi-fluorescence on an Olympus BX61 microscope with a standard rhodamine cube (545 nm and 590 nm of excitation filter and emission filter, Olympus). The fluorescence of white matter was quantified using Image J software (NIH). Five sections were randomly selected from the injury zone. Six replicates were conducted in this test.

#### Statistical analysis

The data is expressed as a mean ± SD. One-way ANOVA were used for statistical analyses (SAS 9.2, SAS Institute Inc., NC, USA). Results showing significance between different chitosan treated groups were subjected to the Tukey’s test. In vivo evaluations used a test of proportions; Fisher’s Exact Test.

## Competing interests

The authors declare they have no competing interests. There is no conflict of interest of any sort in the reporting of these data relative to any author.

## Authors’ contributions

BC drafted the manuscript, YC and RBB designed the experiments. DB and BC performed the experiments. RBB is the Principle Investigator and Director of the CPR and is responsible for all elements of the research. This work is in partial fulfillment of the Doctoral Degree requirements of BC. All authors read and approved final manuscript.

## References

[B1] Luo J Li N Robinson JP Shi R The increase of reactive oxygen species and their inhibition in an isolated guinea pig spinal cord compression model Spinal Cord 2002 40 656 665 10.1038/sj.sc.3101363 12483500

[B2] Borgens RB Shi R Immediate recovery from spinal cord injury through molecular repair of nerve membranes with polyethylene glycol FASEB J 2000 14 27 35 10627277 10.1096/fasebj.14.1.27

[B3] Borgens RB Cellular engineering: molecular repair of membranes to rescue cells of the damaged nervous system Neurosurgery 2001 49 370 378 discussion 378–379 11504113 10.1097/00006123-200108000-00021

[B4] Schwab ME Repairing the injured spinal cord Science 2002 295 1029 1031 10.1126/science.1067840 11834824

[B5] Cho Y Borgens RB Polymer and nano-technology applications for repair and reconstruction of the central nervous system Exp Neurol 2012 233 126 144 10.1016/j.expneurol.2011.09.028 21985867

[B6] Borgens RB Liu-Snyder P Understanding secondary injury Q Rev Biol 2012 87 89 127 10.1086/665457 22696939

[B7] Shi Y Kim S Huff TB Borgens RB Park K Shi R Cheng JX Effective repair of traumatically injured spinal cord by nanoscale block copolymer micelles Nat Nanotechnol 2009 5 80 87 19898498 10.1038/nnano.2009.303 PMC2843695

[B8] Liu-Snyder P Borgens RB Shi R Hydralazine rescues PC12 cells from acrolein-mediated death J Neurosci Res 2006 84 219 227 10.1002/jnr.20862 16619236

[B9] Liu-Snyder P Logan MP Shi R Smith DT Borgens RB Neuroprotection from secondary injury by polyethylene glycol requires its internalization J Exp Biol 2007 210 1455 1462 10.1242/jeb.02756 17401128

[B10] Borgens RB Bohnert D Rapid recovery from spinal cord injury following subcutaneously administered polyethylene glycol J Neurosci Res 2001 66 1179 1186 10.1002/jnr.1254 11746451

[B11] Koob AO Colby JM Borgens RB Behavioral recovery from traumatic brain injury after membrane reconstruction using polyethylene glycol J Biol Eng 2008 2 9 10.1186/1754-1611-2-9 18588669 PMC2474576

[B12] Koob AO Duerstock BS Babbs CF Sun Y Borgens RB Intravenous polyethylene glycol inhibits the loss of cerebral cells after brain injury J Neurotrauma 2005 22 1092 1111 10.1089/neu.2005.22.1092 16238486

[B13] Donaldson J Shi R Borgens R Polyethylene glycol rapidly restores physiological functions in damaged sciatic nerves of guinea pigs Neurosurgery 2002 50 147 156 11844245 10.1097/00006123-200201000-00023

[B14] Brent J Current management of ethylene glycol poisoning Drugs 2001 61 979 988 10.2165/00003495-200161070-00006 11434452

[B15] Caravati EM Erdman AR Christianson G Manoguerra AS Booze LL Woolf AD Olson KR Chyka PA Scharman EJ Wax PM Ethylene glycol exposure: an evidence-based consensus guideline for out-of-hospital management Clin Toxicol (Phila) 2005 43 327 345 10.1080/07313820500184971 16235508

[B16] Schneiderman S Farber JL Baserga R A simple method for decreasing the toxicity of polyethylene glycol in mammalian cell hybridization Somat Cell Mol Genet 1979 5 263 269 10.1007/BF01539165 384568

[B17] Szabó G Kiss A Trón L Permeabilization of lymphocytes with polyethylene glycol 1000. Discrimination of permeabilized cells by flow cytometry Cytometry 1982 3 59 63 10.1002/cyto.990030113 6180875

[B18] Webster R Elliott V Park BK Walker D Hankin M Taupin P Veronese FM PEG and PEG conjugates toxicity: towards an understanding of the toxicity of PEG and its relevance to PEGylated biologicals PEGylated Protein Drugs: Basic Science and Clinical Application 2009 Switzerland: Birkhäuser Basel 127 146 Milestones in Drug Therapy

[B19] Cole A Shi R Prolonged focal application of polyethylene glycol induces conduction block in guinea pig spinal cord white matter Toxicol Vitr 2005 19 215 220 10.1016/j.tiv.2004.10.007 15649635

[B20] Borgens RB Restoring function to the injured human spinal cord 2003 Heidelberg, Germany: (Monograph) Springer-Verlag 12793206

[B21] Cho Y Shi R Borgens RB Chitosan produces potent neuroprotection and physiological recovery following traumatic spinal cord injury J Exp Biol 2010 213 1513 1520 10.1242/jeb.035162 20400636

[B22] Chavasit V Torres JA Chitosan-poly(acrylic acid): mechanism of complex formation and potential industrial applications Biotechnol Prog 1990 6 2 6 10.1021/bp00001a001 1366433

[B23] Lee DW Lim H Chong HN Shim WS Advances in chitosan material and its hybrid derivatives: a review Open Biomater J 2009 1 10 20 10.2174/1876502500901010010

[B24] Aranaz I Mengibar M Harris R Panos I Miralles B Acosta N Galed G Heras A Functional characterization of chitin and chitosan Curr Chem Biol 2009 3 203 230

[B25] Pavinatto FJ Caseli L Pavinatto A Dos Santos DS Jr Nobre TM Zaniquelli ME Silva HS Miranda PB De Oliveira ON Jr Probing chitosan and phospholipid interactions using Langmuir and Langmuir-Blodgett films as cell membrane models Langmuir 2007 23 7666 7671 10.1021/la700856a 17539668

[B26] Pavinatto FJ Pavinatto A Caseli L Santos DS Jr Nobre TM Zaniquelli ME Oliveira ON Jr Interaction of chitosan with cell membrane models at the air-water interface Biomacromolecules 2007 8 1633 1640 10.1021/bm0701550 17419586

[B27] Pavinatto FJ Caseli L Oliveira ON Chitosan in nanostructured thin films Biomacromolecules 2010 11 1897 1908 10.1021/bm1004838 20590156

[B28] Quemeneur F Rinaudo M Pepen-Donat B Influence of molecular weight and pH on adsorption of chitosan at the surface of large and giant vesicles Biomacromolecules 2008 9 396 402 10.1021/bm700943j 18067258

[B29] Chattopadhyay DP Inamdar MS Aqueous behaviour of chitosan Int J Polym Sci 2010

[B30] Fang N Chan V Mao HQ Leong KW Interactions of phospholipid bilayer with chitosan: effect of molecular weight and pH Biomacromolecules 2001 2 1161 1168 10.1021/bm015548s 11777388

[B31] Fang N Chan V Chitosan-induced restructuration of a mica-supported phospholipid bilayer: an atomic force microscopy study Biomacromolecules 2003 4 1596 1604 10.1021/bm034259w 14606885

[B32] Chen B Zuberi M Borgens RB Cho Y Affinity for, and localization of, PEG-functionalized silica nanoparticles to sites of damage in an ex vivo spinal cord injury model J Biol Eng 2012 6 18 10.1186/1754-1611-6-18 22979980 PMC3549791

[B33] Lentz B PEG as a tool to gain insight into membrane fusion Eur Biophys J 2007 36 315 326 10.1007/s00249-006-0097-z 17039359

[B34] Shi R Borgens RB Acute repair of crushed guinea pig spinal cord by polyethylene glycol J Neurophysiol 1999 81 2406 2414 10322076 10.1152/jn.1999.81.5.2406

[B35] Cho Y Shi R Borgens R Ivanisevic A Repairing the damaged spinal cord and brain with nanomedicine Small 2008 4 10 1676 1681 10.1002/smll.200800838 18798208

[B36] Cho Y Shi R Ben Borgens R Chitosan nanoparticle-based neuronal membrane sealing and neuroprotection following acrolein-induced cell injury J Biol Eng 2010 4 2 10.1186/1754-1611-4-2 20205817 PMC2824642

